# Composite of Elastin-Based Matrix and Electrospun Poly(L-Lactic Acid) Fibers: A Potential Smart Drug Delivery System

**DOI:** 10.3389/fbioe.2018.00127

**Published:** 2018-09-12

**Authors:** Antonella Bandiera, Sabina Passamonti, Luisa Stella Dolci, Maria Letizia Focarete

**Affiliations:** ^1^Department of Life Sciences, University of Trieste, Trieste, Italy; ^2^Department of Chemistry “G. Ciamician” and National Consortium of Materials Science and Technology (INSTM, Bologna RU), Alma Mater Studiorum - Università di Bologna, Bologna, Italy; ^3^Health Sciences and Technologies–Interdepartmental Center for Industrial Research, Alma Mater Studiorum - Università di Bologna, Bologna, Italy

**Keywords:** elastin, electrospun matrix, composite, smart release, drug delivery

## Abstract

Stimuli-responsive hydrogel matrices are inspiring manifold applications in controlled delivery of bioactive compounds. Elastin-derived polypeptides form hydrogel matrices that may release bioactive moieties as a function of local increase of active elastases, as it would occur in several processes like inflammation. In view of the development of a patch for healing wounds, recombinant elastin-based polypeptides were combined with a proteolysis-resistant scaffold, made of electrospun poly-L-lactic acid (PLLA) fibers. The results of this study demonstrated the compatibility of these two components. An efficient procedure to obtain a composite material retaining the main features of each component was established. The release of the elastin moiety was monitored by means of a simple protocol. Our data showed that electrospun PLLA can form a composite with fusion proteins bound to elastin-derived polypeptides. Therefore, our approach allows designing a therapeutic agent delivery platform to realize devices capable of responding and interacting with biological systems at the molecular level.

## Introduction

The biomaterial field has enormously evolved in the last decades. Many different materials have been developed and are available to realize devices that can be used for a wide variety of applications. With the advent of bioengineering and regenerative medicine, the field of biomaterials entered the phase of developing devices capable of actively interacting with the biological system, rather than passively integrating within it (Hench and Polak, 2002). However, despite the huge work done in this field, there is a constant demand for innovative solutions in order to address many still unmet biomedical needs (Holzapfel et al., 2013).

Among the many composite biomaterials that have been developed and tested for medical applications, the combination of electrospun fibers and hydrogels recently attracted the attention of researchers. Due to their individual features, combining the advantages of both components results in a product with superior properties, that has a high potential for expanding the range of applications of the final construct (reviewed in Bosworth et al., 2013; Xu et al., 2016).

The advantages achieved by the combination of electrospun fibers and hydrogels have been demonstrated in the field of controlled drug delivery, where controlled release could be obtained by exploiting the release characteristics of the two components (Han et al., 2013; Bruggeman et al., 2017). In the field of tissue engineering, combining the biomimetic properties, hydrophilicity, and softness of hydrogels with the mechanical strength of electrospun sheets, allows to mimic the structure of tissue extracellular matrix (Gualandi et al., 2016). Other fields of application of electrospun fibers/hydrogels composites are those of biotechnology and biosensors (Xu et al., 2016).

Electrospun fibrous mats, made of natural or synthetic polymers, exhibit high porosity, high surface area to volume ratio, and good mechanical properties. Moreover, these properties can be easily tailored by changing the fiber diameter through proper control of the electrospinning process. For these reasons, electrospun mats represent a valuable platform for drug delivery and tissue engineering and regeneration (Chen et al., 2018).

Elastin-like polypeptides are an emerging class of biotechnologically derived biopolymers that are inspired to the tissue structural protein elastin (MacEwan and Chilkoti, 2010; Girotti et al., 2011). In our lab, starting from design, cloning and expression of synthetic genes, a family of recombinant proteins named Human Elastin-like Polypeptides (HELPs) was produced (Bandiera, 2010). This versatile platform can be readily customized by the fusion of bioactive domains of interest, thus embedding the new functionality in the final construct. A method for the preparation of hydrogel matrix based on these HELPs was set up (Bandiera, 2011) and the specific stimuli-induced release was demonstrated (Bandiera et al., 2014).

To the best of our knowledge, there are only few examples of elastin-like based composites that have been developed till now (reviewed in Kakinoki et al., 2014; Yeo et al., 2015). Here, we describe an approach to obtain a new composite material based on deposition of elastin-like based on electrospun poly-L-lactic acid (PLLA-HELP).

## Materials and methods

### HELP biopolymers

HELP and mHELP, the latter being a construct of HELP obtained by fusing at the C-terminal region a functional domain (unpublished data) were produced exploiting their inverse phase transition properties as already detailed (Bandiera, 2010). The purified products were checked by SDS-PAGE and lyophilized.

### Electrospun PLLA scaffold fabrication

Poly(L-lactic acid) (PLLA) (Lacea H.100-E) (Mw = 8.4 10^4^g mol^−1^, PDI = 1.7) was supplied by Mitsui Fine Chemicals. Dichloromethane (DCM) and dimethylformamide (DMF), were purchased by Sigma–Aldrich and were used without any further purification. PLLA was dissolved in a mixed solvent, DCM:DMF = 65:35 v/v, at a concentration of 13% w/v. The polymeric solution was electrospun by means of an electrospinning apparatus (Spinbow srl, Italy) by applying the following processing conditions: applied voltage = 18 kV, feed rate = 0.015 ml min^−1^, needle-to-collector distance = 15 cm. The electrospun mat was produced at RT and at relative humidity of 40–50% and was kept under vacuum over P_2_O_5_ at RT overnight in order to remove residual solvents.

### Water contact angle (WCA) measurements

Static WCA measurements were performed at RT under ambient conditions by using an optical contact angle and surface tension meter KSV's CAM 100 (KSV, Espoo, Finland). Milli-Q water was used for measurements. The water drop profile images were collected in a time range of 0–60 s, every 1 s. Sixty seconds was selected as the upper time limit since it was verified that, after that period, the WCAs reached a constant value. Optical contact angle and pendant drop surface tension software was used for image processing. Results (WCA at 60 s) were averaged on at least five measurements obtained at different areas of the sample.

### HELP deposition and cross-linking on PLLA

Five percent (w/v) of water solution of HELP and the other proteins tested were prepared and 5 μl of each sample were spotted on small sheets of PLLA mat. Water was evaporated at RT and the dried samples were stored (controls) or washed with excess water, respectively. Washed samples were dried as well. For cross-linking, 2 μl of microbial transglutaminase (60 mg/ml, N-Zyme Biotec GmbH, Darmstadt, Germany) were added to 30 μl of 5% (w/v) HELP or its fusion in 10 mM Tris/HCl pH 8 (Sigma-Aldrich, #T1503). Spots of 5 μl were deposited on PLLA mat and the reaction was carried on for 1 h at RT or at 5°C overnight in a wet chamber to avoid drying. After cross-linking the samples were washed overnight as described above and stored dry.

### Evaluation of protein retention on PLLA

Samples of PLLA with adsorbed or cross-linked HELP were stained for 10 min in 0.5 mg/mL Amidoblack (Serva, #12310), 50% Ethanol and then rinsed twice with water for 10 min. Each spot was cut off and soaked in 200 μl of 50 mM Tris/HCl pH 7.5, 1 mM CaCl_2_, in the absence or in presence of 0.5 μg elastase (Sigma-Aldrich, #E7885). Samples were incubated overnight at 37°C. Supernatants were read at 620 nm by a microplate reader (Synergy H1, Bio Tek).

### Scanning electron microscopy (SEM) analysis

After cross-linking samples were rinsed with excess water. They were frozen at −20°C and lyophilized. Slices were cut, mounted onto stubs using a double-sided adhesive and sputter coated with gold. Analysis was performed using a Leica Stereoscan 430i Scanning Electron Microscope.

## Results

**Figure 1:**
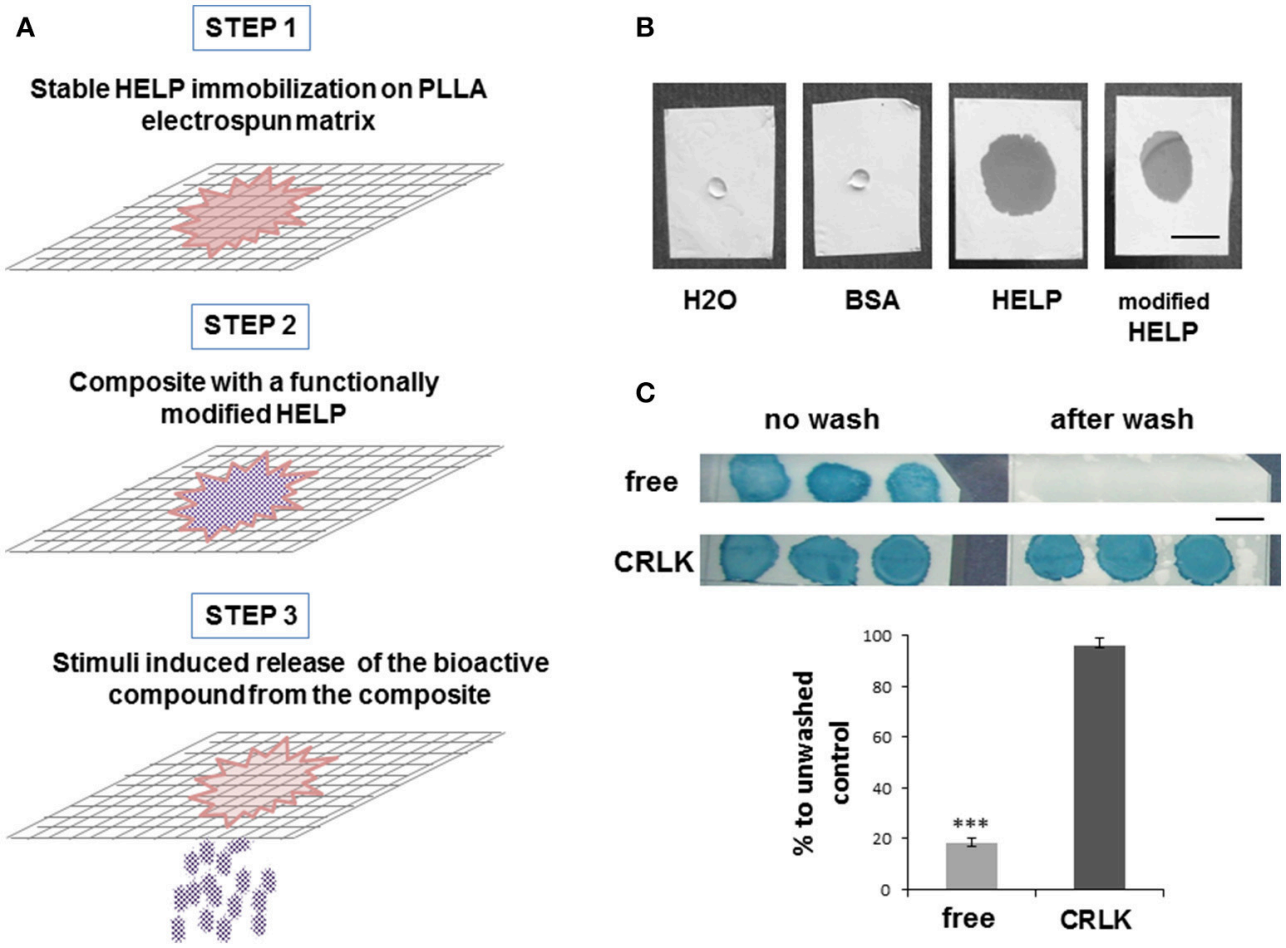
HELP-PLLA composite design. **(A)** Schematic description of the experimental approach; **(B)** Representative images of different protein aqueous solutions 0.5 min after deposition on PLLA matrix. Bar is 5 mm; **(C)** Stabilization effect of the HELP cross-linking process (CRLK) compared to simple deposition of the protein (free) on PLLA matrix: representative images of unwashed and washed samples, Amidoblack staining. Bar is 5 mm. Evaluation of sample stability after washing is reported in the histogram. Each value represented the mean ± SD, *n* = 3. Differences between free and cross-linked samples after extensive washing were assessed by one-way ANOVA, ***p < 0.001 compared to unwashed samples.

Our strategy to prepare a PLLA-HELP composite is schematized in Figure 1A.

The approach used in this study is the physical combination of two materials, through deposition of HELP-based protein onto the surface of PLLA mat. Blending of HELP protein and PLLA solution before electrospinning was not possible since the organic solvents required for electrospinning PLLA were not compatible with the protein. Moreover, only a small amount of the protein would have been available at the surface of the fibers. The first step of composite fabrication consisted in the evaluation of the compatibility of these two materials, since WCA measurement demonstrated that electrospun PLLA is a hydrophobic material (WCA = 120° ± 3°), whereas the HELP protein is soluble in aqueous solution. Both HELP and one of its fusion products were dissolved in water and dropped on a PLLA sheet, avoiding the physical contact with any surface beneath it. A solution of Bovine Serum Albumin (BSA) at the same concentration was tested as reference. Interestingly, as shown in Figure 1B, PLLA samples in contact with HELP-based protein solutions immediately became wet and the drop spread over the sheet, whereas when a drop of BSA was deposited onto the PLLA sheet, no wetting was observed. After water evaporation, the sample deposited on PLLA was no longer observable. To assess the presence of HELP protein on PLLA, we stained the samples with Amidoblack. After de-staining, the protein became evident (Figure 1C, top left).

In parallel, a replica sample was prepared and submitted to an overnight wash before the staining procedure. As shown in Figure 1C (top right), almost no stain was detectable after the overnight wash. On the contrary, when the transglutaminase enzyme was added to the HELP protein to determine its cross-linking (Bandiera, 2011), no difference could be detected between the unwashed (Figure 1C, bottom left) and the overnight washed replica (Figure 1C, bottom right). This indicated that the enzymatic cross-linking stabilized the HELP protein on the PLLA sheet.

We set up a method to estimate the stability of HELP deposited on PLLA, by exploiting the susceptibility of HELP to protease-dependent elastolysis (Corich et al., 2017). Figure 1C shows that after the wash, cross-linked HELP-PLLA samples retained up to 96% of the HELP protein after the wash step, whereas < 20% of it remained in the non-cross-linked samples. This suggested that cross-linking caused the formation of a stable HELP-PLLA composite.

Since this work was undertaken with the aim of realizing smart devices endowed with environmentally-controlled functionality, it was of great interest to assess if fusion-modified HELP could be stabilized in the PLLA matrix as well. For this reason, mHELP, a fusion-modified HELP that we recently obtained by cloning a functional binding domain to the HELP backbone (unpublished results), was employed. The same procedure used for HELP-PLLA composite was applied to mHELP-PLLA samples and the analyses were performed giving results similar to those shown in Figure 1C (not shown).

**Figure 2:**
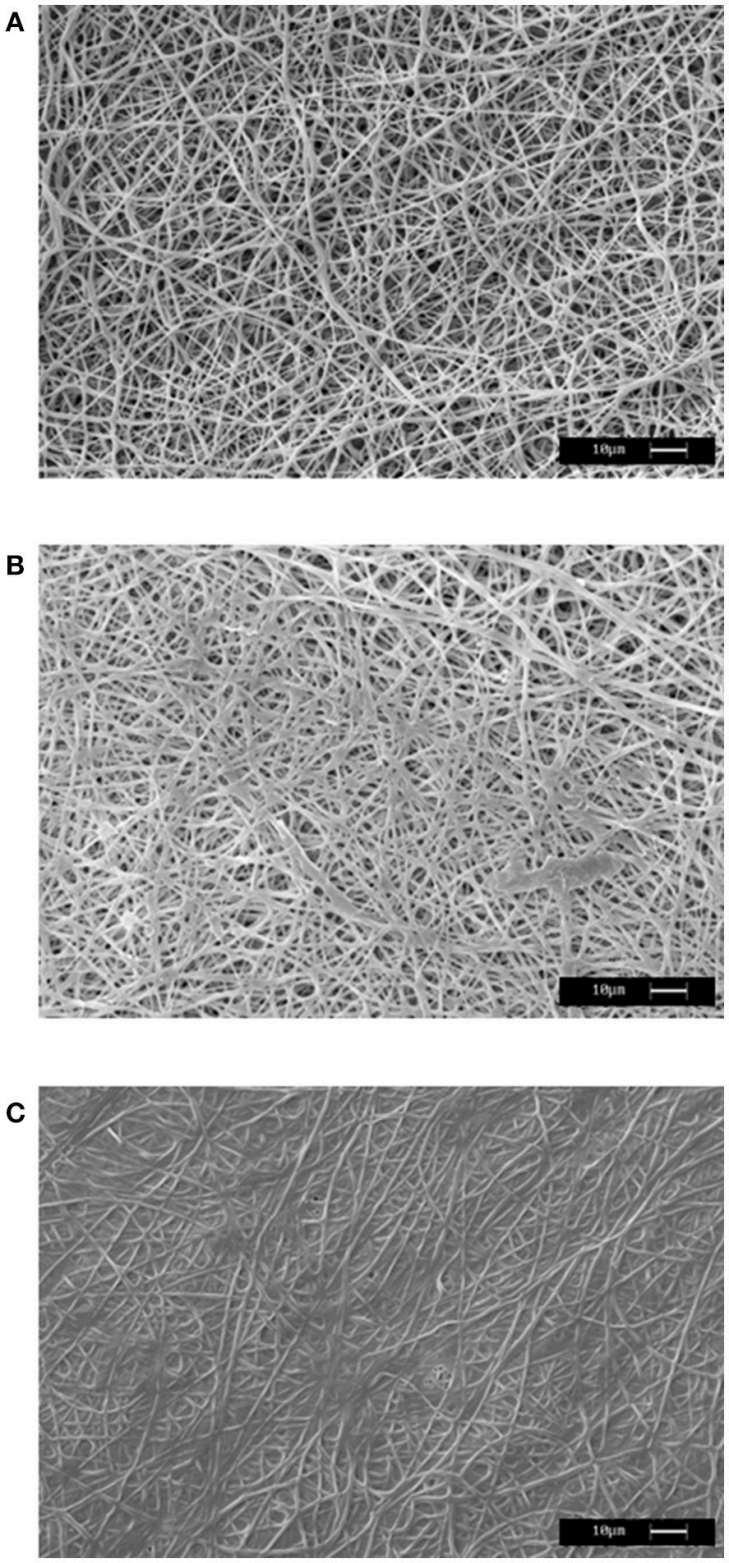
Representative SEM images of PLLA mat **(A)**, mHELP deposited on the PLLA matrix in the absence of cross-linking **(B)** and mHELP undergone cross-linking after deposition on PLLA matrix **(C)**. Bar is 10 μm.

To obtain further information on the morphology of the stabilized mHELP-PLLA samples, SEM analysis was performed. Figure 2A shows SEM analysis of PLLA electrospun fibers. Cross-linking the composite, even after extensive overnight washing, changed the morphology of PLLA, which appeared as coated by a continuous, amorphous layer, filling the pores among the fibers (Figure 2C). In the absence of cross-linking, tiny deposits of protein were observed on PLLA only after a quick wash (Figure 2B).

These results indicate that both HELP macromolecule and its fusion modifications could be employed for preparation of new composites endowed with functional activity. Analysis of activity retention is currently ongoing.

## Discussion

HELP matrix has been shown to possess stimuli-responsive properties, undergoing selective degradation in the presence of elastolytic activity. Exploiting this feature represents an attractive option to realize smart systems that can be employed for therapeutic delivery, tissue engineering, and biosensing (Corich et al., 2017).

In this view, we explored the possibility of integrating the HELP hydrogel matrix with an electrospun PLLA support and our approach is schematized in Figure 1A. Besides being endowed with adequate mechanical tensile strength, electrospun PLLA has a high degree of biocompatibility and has been extensively employed in the fabrication of bioresorbable scaffolds for tissue engineering applications (Chen et al., 2018). Electrospun PLLA is known to be hydrophobic. To increase its hydrophilicity and wettability, surface modifications with functional groups are needed. These enable conjugation or chemical interactions with hydrogels (Dolci et al., 2014; Gualandi et al., 2016). In our approach, the first step of composite fabrication consisted in the evaluation of the compatibility and in the exploration of the conditions for a stable integration of the HELP-based hydrogel in the PLLA electrospun mat. Interestingly, we found that no treatment was needed to enhance PLLA wettability for HELP deposition. The presence of the protein in solution allowed the instantaneous PLLA fiber permeation by the solution (Figure 1B). The finding that physical adsorption of HELP-based proteins onto PLLA mat increased the wetting properties of electrospun PLLA surface is in agreement with previous studies on substrates modified with other elastin-based molecules (Jordan et al., 2007; Srokowski and Woodhouse, 2013) and it is likely related to the relatively high hydropathy index of the HELP protein (Bandiera et al., 2010). Notably, the cross-linking process was the key step to stabilize the HELP moiety on the PLLA mat (Figure 1C).

Keeping in mind that the HELP macromolecule can be tailored by addition of a bioactive domain, in principle, these conditions could be extended to any HELP derivative, obtained by C-terminal domain fusion. Thus, the resulting composite will be endowed with a new, specific functionality. Indeed, in this work we demonstrated that similar result could be obtained using both a HELP protein and mHELP, a fusion-modified HELP, in which the fusion domain represents about one-fifth of the whole macromolecule. SEM analysis was used to achieve the structural evidence of the integration of both materials in a new composite obtained after the cross-linking process (Figure 2C).

This achievement represents the second step of our strategy, i.e., the realization of a composite with a tailored HELP fusion, endowed with a specific functionality (Figure 1A).

A third step can be foreseen, consisting in the stimuli-induced release of the active domain. Future work will be dedicated to fully characterize the new material that opens the way for the realization of stimuli-responsive biomedical devices for the delivery of therapeutic and bioactive substances and for regenerative medicine, as well as for the development of biosensors.

## Conclusion

In this work we explored the opportunity to realize a composite material made of HELP-based hydrogels and PLLA electrospun fibers. The results clearly show that the fabrication of a composite is feasible and that the features of the two materials can be successfully integrated. This strategy is useful to combine the individual performances of the two constituents and future study will clarify the properties of the new hybrid material. The stimuli-responsive nature of the HELP moiety has been already proven and described (Bandiera et al., 2014; Corich et al., 2017) and it represents an advantage that can be conferred to any new material derived from it. Moreover, HELP-based proteins represent a platform that is readily customizable by molecular fusion of exogenous domains, to confer specific functionality to the final product.

We believe that this strategy will contribute to bypass shortcoming and to improve the performance of the single components, opening the way to the realization of devices potentially able to release bioactive compounds upon specific stimuli.

## Author contributions

All the authors conceived and planned the experiments. AB and LD carried out the experimental work. All authors discussed the results and contributed to the final manuscript, reading, and approving the submitted version.

### Conflict of interest statement

The authors declare that the research was conducted in the absence of any commercial or financial relationships that could be construed as a potential conflict of interest.
